# Medical and Physical Intelligence

**Published:** 1824-08

**Authors:** 


### MEDICAL AND PHYSICAL INTELLIGENCE.

### MORBID ANATOMY.

1. *Rupture of the Heart, and Perforation of the Stomach.*—Dr. Andral, jun., at a late meeting of the Academy of Medicine, read the case of a person who died of the above disease: he had suffered a long time from indigestion, and died suddenly from the effect of strong emotions. The pericardium contained a great quantity of blood; the posterior part of the left ventricle presented five oblong perforations, the greatest diameter of which were in the direction of the longitudinal axis of the heart. The heart was not softened in any degree. The stomach showed traces of chronic inflammation, and exhibited, about the centre of its posterior surface, a large circular opening, with soft and round edges. There was no effusion in the peritoneum.—(*Revue Med.* Mars.)

2. *Organic Alterations in the Bronchiæ.*—Dr. Andral, jun., in a Memoir upon this subject, describes three species of alteration to which the bronchiæ are liable,—dilatation, narrowing, and obliteration. Five cases are given of the dilatation of this organ, and from which the following conclusions are drawn:—

1. This dilatation is always connected with the existence of chronic catarrh. 2. There exist three varieties of dilatation: in the first variety, one or more of the bronchiæ present throughout their extent an augmentation of their capacity, more or less considerable; sometimes one branch only is affected, sometimes those of the whole lobe; and the parietes of the canal are often thicker than natural. There is a sort of hypertrophy of the mucous membranes, and even of the cartilaginous tissue itself: if this alteration has not extended far, it is not easily recognized during life. The second variety consists of a swelling of the bronchial canal in one point, of a greater or less extent, with a thickening of its sides: the diagnostic is more or less easy, according to the situation and extent of the enlargement. The third variety is when one of the bronchia is dilated by intervals, presenting a series of successive swellings and contractions: here the sides of the canal, instead of being thickened, are, on the contrary, remarkably thin. It is impossible to establish the diagnostic in this instance.

Contraction of the bronchiæ is a less common alteration than their dilatation, and M. Andral gives but three instances of it. Like the latter complaint, the contraction may be either general or partial. It is not always occasioned by chronic inflammation, but may be the result of mechanic pressure, from tumors, &c. It may exist without producing any phenomena; at others, it produces remarkable symptoms, but which are also common to other affections. In two cases mentioned by M. Andral, the contraction was occasioned by the thickening of the sides of the canal; in the third, the contracted bronchiæ was comprised in an enormous mass of melanosis.

The obliteration of the bronchiæ is an extremely rare occurrence.

M. Andral possesses but one case in which it occurred. The upper lobe of the lung was changed into melanosis; the principal bronchia of this lobe, and its three or four chief divisions, were sound; but beyond that there was no trace of bronchial canals.—*(Archives Generales de Medecine.)*

---

PHYSIOLOGY.

3. *Exhalation of Water in the Act of Respiration.*—Dr. PAOLI and Professor REGNIOLI have had an opportunity of ascertaining the disputed point, whether the water exhaled in the act of respiration came from the lungs, or was owing to the exhalation formed in the aërial and nasal passages, as has been asserted by M. MAGENDIE. Theresa A— had undergone the operation of tracheotomy, and it was observed that the air passing from the wound in the trachea, through a canula, became visible by the condensation of the aqueous vapour, at 4° of Reaumur. A glass was applied four inches distant from this canula, and was covered with moisture.

M. Paoli enters into long discussions on the hypotheses usually advanced on this subject, and comes to the following conclusions:—1st. That the aqueous vapour which accompanies the act of breathing, is formed from the whole surface of the respiratory organs. 2d. That it takes place from simple exhalation, from the mucous membrane investing these organs. 3d. That all the oxygen gas consumed in respiration is employed in the production of the carbonic acid. 4th. That the formation of this acid begins in the lungs, goes on in the arteries and in the circulation, is brought to the lungs with the venous blood; and that by this means the animal heat, produced by the combination of oxygen with the carbon of the blood, is extended to the whole animal economy.

---

PATHOLOGY.

4. *On the Cholera Morbus of India.*—M. MOREAU DE JONNES, who has been occupied during the last five years with researches upon the subject of this disease, has come to the following conclusions respecting it:—

1st. That, from the year 1817 to 1823, it has travelled from the Mollucca Islands to the coasts of Syria,—from the mouth of the Volga, in the Caspian Sea, to the Isles of France and Bourbon; the extreme points of its ravages being 1340 leagues asunder north and south, and 1900 leagues from east to west.

2d. It does not depend upon individual predisposition, since it attacks equally all ages, both sexes, all kinds of temperaments, and different races of mankind.

3d. It does not depend upon the extremes of atmospheric temperature, since its ravages are equally severe at all seasons.

4th. It is not the effect of humidity, or of low and inundated situations; for it has established itself, with equal violence, in the mountains of Nepaul, in the elevated spots of the Isle of France, in the sands of Arabia, the deserts of Diarbekir, and the stappes of Tartary.

5th. It is not produced by marsh miasmata, stagnant water, or other causes of this kind; because it is found in situations where none of these causes exist.

6th. It is not dependent upon any vitiated state of the atmosphere; for it has shown itself with equal malignancy at the opposite extremities of Asia, during a period of seven years.

7th. It is not the consequence of poor or defective nourishment, such as the fish of the Ganges, or the rice of Oude; for it attacks those whose diet is totally different.

8th. It is not transported by the winds, as has been supposed, for many reasons, but especially as it spreads often in a direction contrary to the prevailing currents of air.

9th. These negative propositions lead to the conclusion, that this disease has no connexion with cholera morbus, properly so called; but that it is a pestilential malady, propagated from person to person, but following laws of its own, not yet perfectly known: and, finally, that it has spread from individual to individual, by navigation, by following the march of armies, or of the native pilgrims, or attending upon vessels of war and commerce; crossing the sea with navigators, the desert with caravans, and chains of mountains with travellers.

---

### THERAPEUTICS.

5. *Utility of Pulvis Antimonialis.*—We have been favoured with a communication relative to the powers of this remedy, by Mr. F. Moore, many years chemical operator at Apothecaries' Hall. The following are extracts from his letter:—

"Within the last thirty years,—I mean during the time that I was chemical operator to the Society of Apothecaries,—I not only superintended, but have sweated myself in the preparation of very large quantities of the pulvis antimonialis, as directed in the London Pharmacopœia. Being a very favourite medicine of mine, I have also given a great deal of it; and have always found that, either when taken alone or conjointly with other medicines, it almost constantly either nauseates, vomits, sweats, or acts on the bowels, producing these effects with a dose of from two to five grains: this has been my experience invariably. As operator, I constantly had the opportunity of hearing of it through the very first practitioners; and, during the last long war, the Company had large orders frequently from the different government hospitals: for instance, from Haslar, often for four hundred ounces at a time. Surely, if this medicine were then so much admired and in demand, (and I believe is now exactly, if not better, prepared,) it must still be capable of producing all the good effects expected from it as a medicine, without giving drachm doses. The inference I would draw, then, is, that both Drs. Elliotson and F. Hawkins have not employed the same pulvis antimonialis that I have alluded to. In my time I have heard of extraordinary doses of tartar emetic being given to vomit,—perhaps, from seven to ten grains; but no practitioner, using what is made at the Hall, would, I should think, order more than from two to five grains,

### SURGERY.

6. *Case of Diplopia, cured by a Surgical Operation.*—Dr. QUADRI, of Naples, relates the case of a man attacked with a species of double vision. After having used all the most energetic remedies in vain, he conceived that the disease was owing to some affection of the optic nerves, and ordered the patient to take Richter's pills. The patient, however, became worse; and, on examining the eye, the iris appeared sound, the movements of the pupil regular and rapid; there was no spectra, or small objects, complained of as floating before the eyes, so common in cases where the nerves are affected. Dr. Quadri conceived the disease to arise from pressure, made by a superabundant quantity of cellular substance at the inner angle of the right caruncle, which threw the eye out of the centre of the orbit; and he was confirmed in that opinion upon finding that the diplopia disappeared when the eye was carried inwards or upwards. The author, therefore, laid open the right caruncle, and, by means of a small pair of forceps, took away three small pieces of cellular substance; the wound healed by the first intention, and the patient was relieved.

This encouraged M. Quadri to repeat the operation some few days after, in the same manner, and to the same extent. After this second operation, the wound was closed with a point of suture and adhesive plaster. The inflammation after this operation was more considerable, but was relieved by leeches, and the wound healed in a few days. The disease was quite cured.

M. Quadri conceives that the inflammation which took place after the last operation, increased the density of the cellular substance, so as to destroy the tendency to grow, which it had before evinced.—(*Osserv. Mediche,* March.)

---

### CHEMISTRY.

7. *Artificial Chalybeate Water.*—If a few pieces of silver coin (says Dr. HARE,) be alternated with pieces of sheet iron, on placing the pile in water, it soon acquires a chalybeate taste and a yellowish hue, and in twenty-four hours flocks of oxide of iron appear. Hence, by replenishing with water a vessel in which such a pile is placed, after each draught, we may obtain a competent substitute for a chalybeate spring.—(*Journal of Science.*)

8. *Liquefaction of Sulphureous Acid.*—M. BUSSY is stated to have obtained the above acid liquid, and free from water, by causing it to pass, in its gaseous state, through a tube containing fused chloride of calcium, and afterwards into a flask surrounded by a mixture of ice and salt, where it completely liquefies, and remains in a liquid state under atmospheric pressure, at the temperature of 0°. It is a colourless, transparent, and very volatile liquid, of a specific gravity = 1.45. It boils at about 10° centigrade below 0 = 14° Fahrenheit; but, in consequence of the cold produced by the evaporation of the portion which is volatilized, the residue remains liquid, being reduced to a temperature much below its boiling point. It occasions intense cold, and rapidly evaporates when dropped upon the hand. Poured into water at common temperatures, one portion is dissolved and another volatilized; but, as the solution approaches to saturation, the acid collects in drops at the bottom of the vessel, like an oil heavier than water. If in this state it be touched by the extremity of a glass tube, it passes into vapour, occasioning ebullition, and ice forms upon the surface of the water.

The bulb of a thermometer enveloped in cotton, and dipped into the liquid acid, falls spontaneously, when exposed to the air, to — 57°, (= — 70° Fahr.) the atmosphere being at 50° F. In the vacuum of the air-pump, a cold of — 68° (= — 90° F.) is thus easily obtained. Mercury, therefore, is easily frozen by the aid of this acid, simply by dipping the bulb of a mercurial thermometer, surrounded with cotton, into it, and agitating the air with it. The experiment succeeds better when a little mercury is put into a cup, with a small quantity of sulphureous acid upon it, and the whole put under the exhausted receiver. By the evacuation of the acid in vacuo, M. Bussy has frozen alcohol of a strength below 33°, (of a specific gravity below .852 at 55°.) By passing chlorine and ammonia through tubes cooled by the evaporation of sulphureous acid, M. B. liquefied those gases; and, by a similar method, cyanogen was obtained in the form of a crystallized solid.—(*Annales de Chimie et Physique*, Mai.)

9. *Test of the Alteration of Solutions by the Contact of Air.*—M. Becquerel remarks, that, if iron be dissolved in nitric acid, and the solution filtered, and two plates of platina, connected with the two extremities of the wire of a galvanoscope, be immersed into the solution, and if one plate be withdrawn, and then re-introduced into the solution, it will produce an electric current passing from this plate to the other; and generally the plate withdrawn from the solution and re-introduced, becomes positively electrical.

The nitrates of copper and lead give similar results; but they do not retain this power, and in the course of a few hours no effects of this kind are observable. Nitrate of zinc does not operate in this manner. Suspecting that the effect was due to the action of air on the film of solution which adheres to the withdrawn plate, the experiment was made in an atmosphere of hydrogen; and then no such results were obtained. M. Becquerel, therefore, attributes the effect to the alteration induced by the air on the portion of solution withdrawn with the plate, and which, when the plate is re-immersed, being dissimilar to the fluid that has not been exposed, determines the current of electricity. The effect of the air, he considers, is probably to convert such portion of deutoxide of azote and proto-nitrate as may have been formed by the action of nitric acid on the metal, into nitrous acid and deuto-nitrate; and that, when this has taken place with all the portions of the solution, the power of producing electrical currents ceases.—(*Ann. de Chim.* xxv. 413.)

### MISCELLANEOUS.

10. *Society of Physicians of the United Kingdom; established in London, June* 17, 1824.—Although medicine has been studied from a very early period, and considerable genius and learning have been employed in its cultivation, yet such is the extreme complication and difficulty of the subject, that its present state still admits of great improvement; to which, perhaps, nothing would more effectually contribute than the intimate union and active co-operation of its professors.

Much may certainly be accomplished by united effort, which individual exertion, however well directed, is unable to effect. While, at the same time, it cannot be doubted that, whatever contributes to the advancement of medical science, must, by increasing its usefulness, add to the dignity of the profession.

Under these impressions, and considering that a great majority of the regular graduates in physic of this country are at present in an isolated state, several physicians practising in London have been induced to associate, and to invite the zealous co-operation, throughout the kingdom, of that part of the profession to which they belong; with a confident hope of facilitating, by these means, the accomplishment of the laudable purposes just mentioned.

It is, therefore, proposed that a Society be established, having principally in view the following objects:—1. The reception and discussion of subjects connected in any manner with the science of medicine. 2. The combined investigation of such points, whether theoretical or practical, as are at present obscure or uncertain, and to the elucidation of which individual labour has hitherto appeared inadequate. 3. The publication of papers furnished by members of the Society, or of those which may be transmitted to them by the profession at large. 4. And, in general, the effecting of whatever may tend to improve the science of medicine, or to advance the interests and dignity of its professors, the regularly educated graduates in physic of the Universities of the United Kingdom.

At a meeting held June the 17th, 1824, at the house of Dr. Shearman, —present, Doctors Temple, Cleverly, Birkbeck, Uwins, Clutterbuck, Hancock, Shearman, Copland, Tweedie, and Roberts, it was resolved unanimously:—

1. That a Society of Physicians be established, for the purposes above stated.

2. That it be called THE SOCIETY OF PHYSICIANS OF THE UNITED KINGDOM.

3. That the Society consist of such persons only as have actually prosecuted the study of medicine in a University, for the period prescribed by its regulations, and who, having subsequently submitted to the usual tests and examinations, have thereby obtained the degree of Bachelor or Doctor of Physic. But members of the London College, whether Fellows or Licentiates, admitted prior to the year 1800, are eligible.

4. That no person be a member of this Society, who is engaged in the actual practice of surgery, pharmacy, or midwifery.

5. That a committee be appointed for the purposes of giving the necessary publicity to these transactions; of receiving communications from the profession; of preparing a system of laws and regulations for the government of the Society; and of performing, in general, whatever may be conducive to its interests, prior to the first general meeting; to which they are to report proceedings, and resign their functions.

6. That the following gentlemen be members of this committee, with the power of making such additions to their number as they may judge convenient:—Drs. Temple, Cleverly, Birkbeck, Uwins, and Clutterbuck.

7. That the first general meeting take place at the house of Dr. Birkbeck, at half-past eight in the evening of the second Thursday in October next. (Signed)

C. J. ROBERTS, *Sec. pro temp.*

Communications on the subject of the Society, to be addressed to Dr. Roberts, No. 20, Earl-street, Blackfriars.

---

### MONTHLY CATALOGUE OF MEDICAL BOOKS.

### METEOROLOGICAL JOURNAL,

*From June 20, to July 19, 1824.*

By Messrs. William Harris and Co. 50, Holborn, London.

| | MOON. | Rain gauge | THERM. | | | BAROM. | | DE LUC'S HYG. | | WIND. | | ATMOSPHERIC VARIATIONS. | | |
|---|---|---|---|---|---|---|---|---|---|---|---|---|---|---|
| | | | 9 A.M. | MAX. | MIN. | 9 A.M. | 10P.M. | 9 A.M. | 10P.M. | 9 A.M. | 10P.M. | 9 A.M. | 2 P.M. | 10P.M. |
| Jun. 20 | | 30. | 52 | 60 | 53 | 29.30 | 29.30 | 74 | 89 | SW | SSW | Fine | Rain | Rain |
| 21 | | | 57 | 63 | 58 | 29.32 | 29.40 | 75 | 80 | SW | W | Rain | Fine | Fine |
| 22 | | | 60 | 68 | 59 | 29.43 | 29.40 | 65 | 75 | WSW | SSW | Fine | | |
| 23 | | .70 | 60 | 60 | 55 | 29.24 | 29.34 | 85 | 90 | SE | NE | Rain | Rain | Rain |
| 24 | | | 57 | 57 | 50 | 29.33 | 29.50 | 90 | 90 | N | W | Cloud. | | |
| 25 | | | 55 | 58 | 52 | 29.62 | 29.80 | 85 | 70 | W | NW | | Cloud. | Cloud. |
| 26 | ● | | 60 | 65 | 60 | 29.94 | 30.00 | 65 | 65 | W | WSW | Fine | Fine | Fine |
| 27 | | | 63 | 68 | 58 | 29.97 | 29.88 | 60 | 62 | SE | ES E | | | |
| 28 | | | 58 | 69 | 59 | 29.90 | 29.84 | 67 | 65 | WSW | SSW | Rain | | |
| 29 | | .25 | 63 | 72 | 65 | 29.65 | 29.70 | 65 | 66 | SSE | NW | | | |
| 30 | | | 65 | 69 | 57 | 29.83 | 29.82 | 67 | 65 | W S W | WSW | Fine | | |
| July 1 | | | 64 | 66 | 55 | 29.82 | 29.70 | 65 | 70 | W | SW | | Fair | Rain |
| 2 | | .43 | 57 | 68 | 58 | 29.50 | 29.52 | 80 | 79 | SW | SW | Rain | Sho'ry | |
| 3 | ☽ | | 63 | 68 | 55 | 29.57 | 29.53 | 65 | 70 | W | NW | Fine | | Cloud. |
| 4 | | | 60 | 67 | 56 | 29.65 | 29.86 | 68 | 69 | W | NW | | | Fine |
| 5 | | | 63 | 66 | 54 | 29.92 | 29.90 | 63 | 69 | WSW | SW | | Fair | Cloud. |
| 6 | | 17 | 57 | 61 | 56 | 29.84 | 29.80 | 70 | 74 | SE | SW | Rain | Rain | |
| 7 | | | 57 | 67 | 54 | 29.83 | 29.83 | 73 | 70 | SW | W | Cloud. | | |
| 8 | | | 57 | 69 | 58 | 29.95 | 29.97 | 65 | 70 | WSW | SW | Fair | Fine | Fine |
| 9 | | | 60 | 70 | 57 | 29.92 | 29.85 | 70 | 70 | SSW | NNW | Cloud. | Sho'ry | |
| 10 | | | 65 | 71 | 57 | 29.87 | 29.96 | 65 | 64 | WNW | NW | Fine | Fine | |
| 11 | ○ | | 65 | 73 | 58 | 30.07 | 30.00 | 60 | 64 | W | W | | | |
| 12 | | | 65 | 75 | 55 | 30.07 | 29.95 | 62 | 62 | SW | WSW | Fair | | Cloud. |
| 13 | | | 67 | 78 | 60 | 30.01 | 30.00 | 65 | 60 | W | SSW | Fine | Fine | Fine |
| 14 | | | 75 | 78 | 60 | 29.90 | 29.80 | 55 | 65 | E | E | | Sho'ry | Th.Sto |
| 15 | | .70 | 63 | 75 | 60 | 29.86 | 30.00 | 73 | 70 | W | W | Cloud. | Fine | Finc |
| 16 | | | 67 | 73 | 57 | 30.04 | 30.04 | 66 | 64 | W | W | Fine | Sho'ry | Cloud. |
| 17 | | | 66 | 70 | 57 | 30.18 | 30.20 | 65 | 64 | ENE | E | | Fine | Fine |
| 18 | | | 60 | 69 | 56 | 30.20 | 30.35 | 60 | 62 | ENE | NE | | | |
| 19 | ☾ | | 62 | 69 | 58 | 30.40 | 30.40 | 58 | 60 | NW | N | | | |

The quantity of rain fallen in the month of June, was 2 inches and 80.100ths.

